# Mechanochemical Degradation of Caffeine and Diclofenac Using Biochar of Fique Bagasse in the Presence of Al: Monitoring by Mass Spectrometry

**DOI:** 10.1021/acsomega.3c03051

**Published:** 2023-10-12

**Authors:** Yaned Milena Correa-Navarro, Gerson-Dirceu López, Chiara Carazzone, Liliana Giraldo, Juan Carlos Moreno-Piraján

**Affiliations:** † Departamento de Química, Facultad de Ciencias Exactas y Naturales, Grupo de investigación Estudios Ambientales en Agua y Suelo, Universidad de Caldas, Manizales, Caldas 170004, Colombia; ‡ Departamento de Química, Facultad de Ciencias, Grupo de investigación en Sólidos Porosos y Calorimetría, Universidad de los Andes, Carrera 1 No. 18 A-12, Bogotá, D.C. 111711, Colombia; § PhysCheMath Research Group, Facultad de Ciencias y Humanidades, Universidad de América, Avda. Circunvalar No. 20-53, Bogotá, D.C. 111711, Colombia; ∥ Laboratory of Advanced Analytical Techniques in Natural Products (LATNAP), Departamento de Química, Facultad de Ciencias, Universidad de los Andes, Carrera 1 No. 18 A-12, Bogotá, D.C. 111711, Colombia; ⊥ Departamento de Química, Facultad de Ciencias, Universidad Nacional de Colombia, Sede Bogotá, Bogotá, D.C. 11001, Colombia

## Abstract

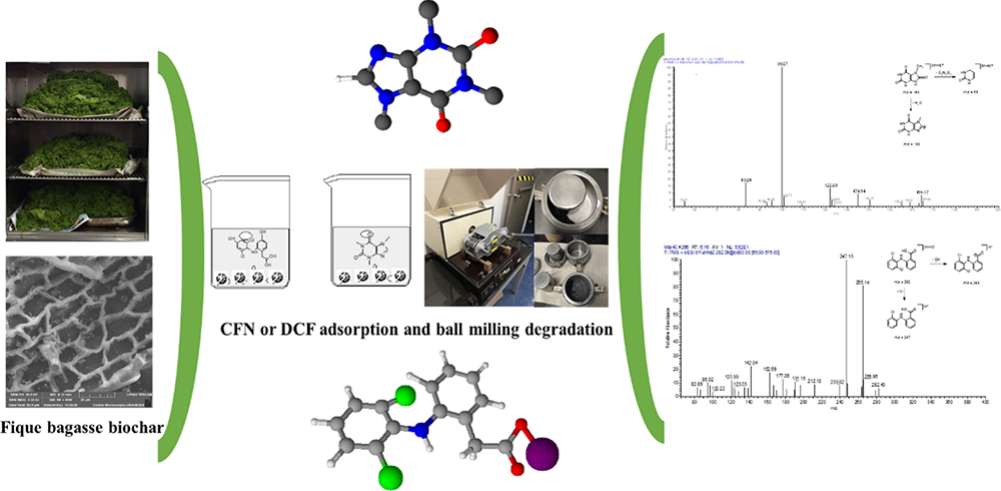

Much research has been carried out to remove emerging contaminants using diverse materials. Furthermore, studies related to pollutant degradation have increased over the past decade. Mechanochemical degradation can successfully decompose molecules that are persistent in the environment. In this study, the biochar of fique bagasse with mixtures SiO_2_, Al, Al_2_O_3_, and Al-Al_2_O_3_ was treated with a mechanochemical technique using a planetary ball mill to investigate the degradation of caffeine and diclofenac. These tests resulted in the transformation of caffeine and diclofenac due to the use of Al employing mechanochemistry. In fact, through the use of liquid chromatography coupled with mass spectrometry, eight and six subproducts were identified for caffeine and diclofenac, respectively. Additionally, analysis of the molecules proposed for caffeine and diclofenac transformation suggested hydroxylation, demethylation, decarboxylation, oxidation reactions, and cleavage of the C–C and C–N bonds in the pollutants studied. The formation of these transformation products could be possible by reductant oxygen species generated from the molecular oxygen in the presence of aluminum and the energy delivered for ball milling. The results obtained show the potential application in the environmental management of mechanochemical treatment in the elimination of emerging contaminants caffeine and diclofenac.

## 1 Introduction

Mechanochemistry (MC) is a field of chemistry that defines the chemical and physical modifications of substances during aggregation caused by the mechanical force obtained through shear, friction, shock, and compression, among others. Consequently, such mechanical energy may disrupt the crystal structure of solids, exposing or creating fresh surfaces rich in active catalytic sites. This mechanical energy delivers the driving force for the mass transfer necessary to bring reactants into contact and induce chemical reactions in the solid state. Pressure or shear stress applied to the impacted particles causes the reactants to become vibrationally and electronically excited, which can facilitate both phase transitions and chemical transformations.^1,2^ Notably, MC was identified by IUPAC as one of the 10 emerging technologies that will change the world and make it sustainable.^3^

Mechanochemical methods have been widely used in extractive metallurgy, materials synthesis, surface modification of solids, and environmental remediation. Compared to conventional procedures, mechanochemical techniques have several advantages which include (a) simplified processes, (b) being environmentally friendly, because of the fact that they are performed in a solid phase, without solvents, at moderate temperatures and pressures, and without combustion, (c) causing less emission of CO_2_ and hazardous byproducts, and (d) it is possible to make a product in a metastable state, which is difficult or impossible to obtain using other conventional methods.^1,4^

For certain mechanochemical processes, reactions can be carried out using the original matrix, for example, unmodified contaminated soil. In addition, to improve the results, coreactants such as inert materials, alkaline earth oxides, and metals, alone or in combination, can also be used. Moreover, due to the mild conditions, the absence of hazardous reagents, and the low ratio of mechanical failures, mechanochemical processes do not have a significant environmental risk.^5^

In environmental remediation (ER), different technologies have been employed to remove contaminants so they can be recycled and subsequently reused as a natural resource.^6^ In fact, ER using mechanochemistry processes is a branch of this technology that has gained interest over the past decade. This new branch is based on the knowledge of inorganic MC, which has been used to synthesize metal alloys and to catalyze their obtention. However, one of the main goals of environmental MC is the degradation of organic pollutants; therefore, research in the field of organic MC has become important.^5^ The first time, MC was used for the degradation of chlorinated pollutants was in 1994.^7^ Since then, progress has been slow, but MC methods are becoming recognized as a noncombustion, fully viable technology for the degradation of organic, inorganic, and emerging pollutants.^8,9^

Currently, MC has gained great relevance; for example, Gobindlal et al. reported the selective degradation of perfluorosulfonic acids (PFSAs) using a mechanochemical process in the presence of SiO_2_. In this work, five PFSA species were degraded with percentages of 99%.^10^ In another research study, the degradation of perfluorooctanoic acid (PFOA) was reached using mechanochemistry with BaTiO_3_. In fact, the removal of PFOAs implied C–F bond cleavage and reduction reactions, mediated by free electrons.^11^ Meanwhile, Yuan et al. reported a mechanochemical process for the elimination of heavy metals (Cu, Pb, and Cd), polybrominated diphenyl ethers (PBDEs), and polychlorinated biphenyls (PCBs) from contaminated soils; in this work, the achieved degradation percentages varied from 85 to 95%.^9^ Likewise, the degradation of thienopyridine from three commercial drugs under MC conditions has been reported in only 15 min of treatment.^12^ According to the results of the studies described above, it can be suggested that mechanochemistry methods can degrade the emerging contaminants: caffeine and diclofenac.

The aim of this work was to study an alternative process to remove emerging contaminants from the environment; in this research, we used adsorption because this technique is environmentally friendly, profitable, and relatively simple.^13^ In addition, fique bagasse biochar was employed as an adsorbent. This carbonaceous material was prepared from waste biomass to add value to this agricultural residue. After adsorption, a mechanochemical process was used to degrade the emerging contaminants caffeine (CFN) and diclofenac sodium (DFC). These are two molecules that have been identified as critical environmental pollutants due to their adverse environmental impact and high persistence in the environment.^14^ In fact, recent studies have shown the hazardous effect of diclofenac on mammals, including humans. Diclofenac could cause gastrointestinal complications, neurotoxicity, cardiotoxicity, hepatotoxicity, nephrotoxicity, hematotoxicity, genotoxicity, teratogenicity, bone fractures, and skin allergy in mammals, even at low concentrations.^15^ On the other hand, it has been evidenced that caffeine exerts adverse impacts on aquatic species and terrestrial insects and can result in a decrease in general stress, induction of oxidative stress, and lipid peroxidation, affecting energy reserves and metabolic activity, neurotoxic effects, affecting reproduction, and even death.^16^ Therefore, cost-effective methods to remove and degrade CFN and DCF from wastewater are urgently needed.

## 2 Materials and Methods

### 2.1 Reagents

Diclofenac sodium was purchased from Sigma-Aldrich. Caffeine, SiO_2_, Al, Al_2_O_3_, and KBr were analytical grade and were supplied by Merck. Formic acid, methanol, and acetonitrile were chromatographic grade and were supplied from Honeywell Fluka (St. Louis, MO, USA).

The procedure for the preparation of the biochar employed in this study was described by Correa et al. in their 2020 study.^17^ To make the biochar, a quantity of fique bagasse was pyrolyzed at 850 °C with a heating rate of 1 °C min^–1^ and residence time for 3 h in an atmosphere of nitrogen; this biochar was labeled as FB850-3. To perform adsorption of emerging contaminants studied, 5.0 mL of CFN or DCF at 25 or 50 mg L^–1^, respectively, was added into a glass vial and put in contact with 20.0 mg of FB850-3; after that, the vessels were placed in an orbital shaker and shaken for 48 h at 20.0 °C. After that, biochar was removed from the vial and dried.

### 2.2 Mechanochemical Analysis

Mechanochemical processing of the fique bagasse biochar plus caffeine or diclofenac at 25 or 50 mg L^–1^, respectively (FB850-3CFN or FB850-3DCF), was carried out in a Hi-speed Vibrating Sample Mill (TI-100CMT, China), equipped with 100 mL sample containers and rod cylindrical-shaped stainless steel. The containers were loaded with 2.000 ± 0.100 g of FB850-3CFN or FB850-3DCF and then were vibration-milled at a rotation speed of 1440 rpm/50 Hz for 1 h at 15 min intervals, at which period of time the container was removed from the mill, and the samples were taken for subsequent evaluation. In addition, the mechanochemical process was also performed with the addition of reagents SiO_2_, Al, Al_2_O_3_, and Al–Al_2_O_3_ (1:1). These experiments were carried out in the same way as previously described, but when the mass of the biochar was put in containers, 6.000 ± 0.100 g of the respective reagent to be tested were also added; consequently, the mass ratio of reagent to biochar was 3:1.^18,19^

After, carrying out the evaluation of the degradation experiments, extractions of all the samples obtained in the mechanochemical processes were performed by placing 0.200 ± 0.010 g of each sample in a glass vial containing 2 mL of methanol. Next, the containers were sonicated for 30 min; subsequently, they were centrifuged at 3000 rpm for 5 min, and then, the supernatant was removed and a second extraction with methanol was performed. Finally, both extracts were combined, filtered through a 0.22 μm PTFE membrane, and analyzed by ultraviolet–visible spectrometry, infrared spectrometry, and high-performance liquid chromatography (HPLC) coupled to ion-trap tandem mass spectrometry (MS^*n*^) according to the methodology described below.

### 2.3 Analytical Methods for the Determination of Caffeine and Diclofenac

Initially, all methanol extracts obtained from mechanochemical assays were evaluated by employing a UV spectrophotometer (UV-1800, SHIMADZU) at λ_max_ = 273 nm for CFN or λ_max_ = 274 nm for DCF. Second, all solids produced by mechanochemical processes of FB850-3CFN and FB850-3DCF were analyzed by Fourier transform infrared spectroscopy (FT-IR) using a Shimadzu IRTracer-100 FT-IR spectrophotometer. In all cases, samples were mixed with KBr and kept in an oven at 105 °C for 24 h prior to analysis. Finally, samples with signals of degradation were analyzed through HPLC-MS^*n*^.

### 2.4 HPLC-ESI-MS^*n*^ Analysis

Samples were analyzed by HPLC-MS^*n*^ using an ultrahigh-performance liquid chromatograph Dionex UltiMate 3000 equipped with a binary pump, online degasser, autosampler, a thermostated column compartment, and diode-array detector (DAD) coupled with an LCQ Fleet Ion Trap Mass Spectrometer (Thermo Scientific, San Jose, CA, USA) through an electrospray (ESI) ion source operated in positive ionization mode. Raw metabolite data were acquired and processed using the Xcalibur 4.3 software (Thermo Scientific, San Jose, CA, USA). Diode array detection was performed over the entire UV–vis range (200–800 nm), and the characteristic absorbances of the CFN and DCF were extracted between 270 and 280 nm.^20^ The RP-HPLC separation was performed at a flow rate of 500 μL min^–1^ with 10 μL injection volume (samples at 5 °C) on a Zorbax SB-C18 column (150 × 4.6 mm i.d., 3.5 μm, Agilent Technologies, Santa Clara, CA, USA) at 40 °C using isocratic separation conditions for 15 min with solvent A: 0.1% formic acid in water and solvent B: 0.1% formic acid in acetonitrile, relation (4:6) respectively.

The identification of the CFN and DCF, as well as their respective degradation products, was performed by MS using the following conditions: source ESI, spray voltage: 4.0 kV; capillary temperature: 275 °C; sheath gas flow rate: 15 (arbitrary units); aux gas flow rate: 5 (arbitrary units); capillary voltage: 19 V; tube lens: 65 V. The ion trap was set to operate in full scan over the range 50–700 mass/charge (*m*/*z*), with acquisition in data-dependent MS/MS and Ion Tree mode with breadth 5 and depth 4 (30% collision energy) to obtain the corresponding fragment ions with an isolation width of 2 *m*/*z*.

## 3 Results

**Figure 1:**
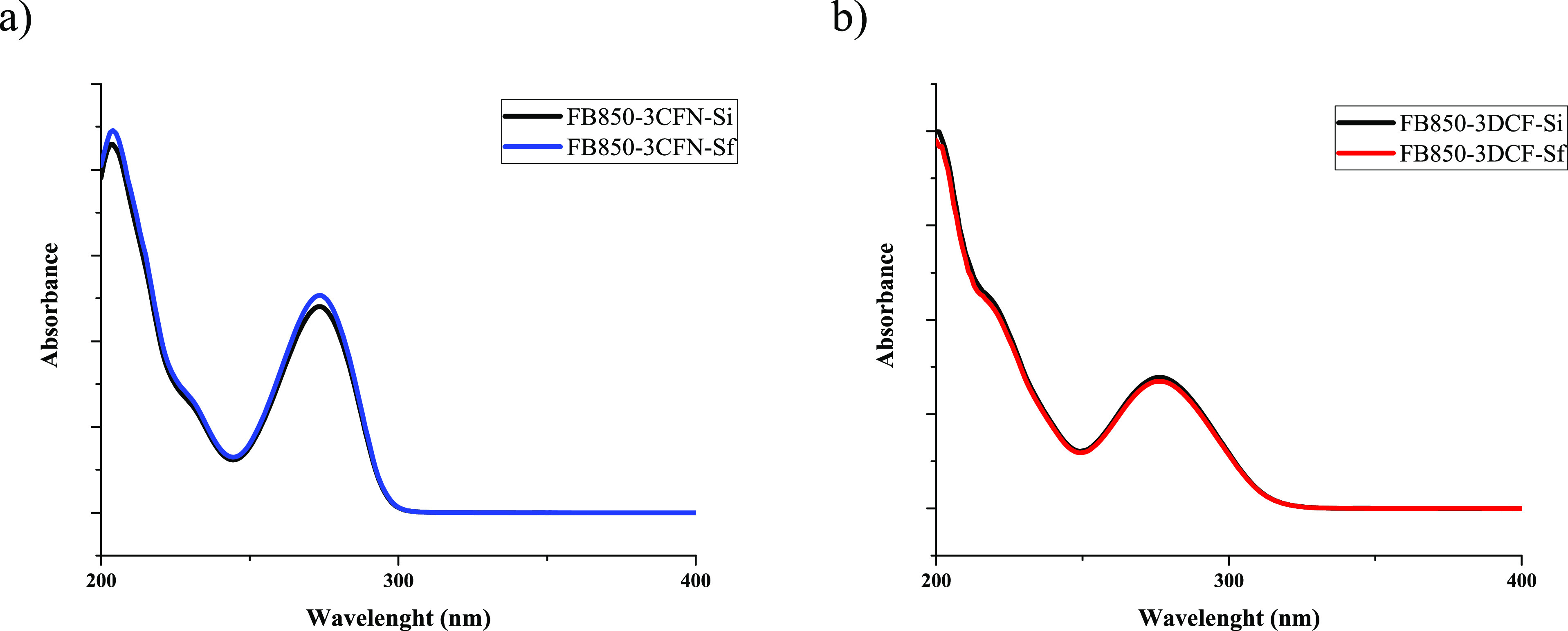
Ultraviolet spectra of methanolic extract of a biochar with caffeine or diclofenac after mechanochemical process: (a) initial and final FB850-3CFN, (b) initial and final FB850-3DCF.

Spectra of methanolic extracts obtained from the FB850-3 + CFN or FB850-3 + DCF milling process showed no differences (Figure 1a,b); therefore, it was considered that the molecules studied had not been transformed in these assays. Despite these results, spectrometric analysis of methanolic extracts after milling, from FB850-3 + CFN or FB850-3 + DCF plus silicon oxide (SiO_2_ a neutral species); aluminum (Al a reducing agent) and aluminum oxide (Al_2_O_3_ a Lewis base), showed promising data but still had inconclusive results; therefore, we carried out an infrared spectrometry analysis. Consequently, our experiments confirm the bibliographic data which states that mechanochemistry techniques improve the process by increasing reactivity with the use of coreagents.^5^

**Figure 2:**
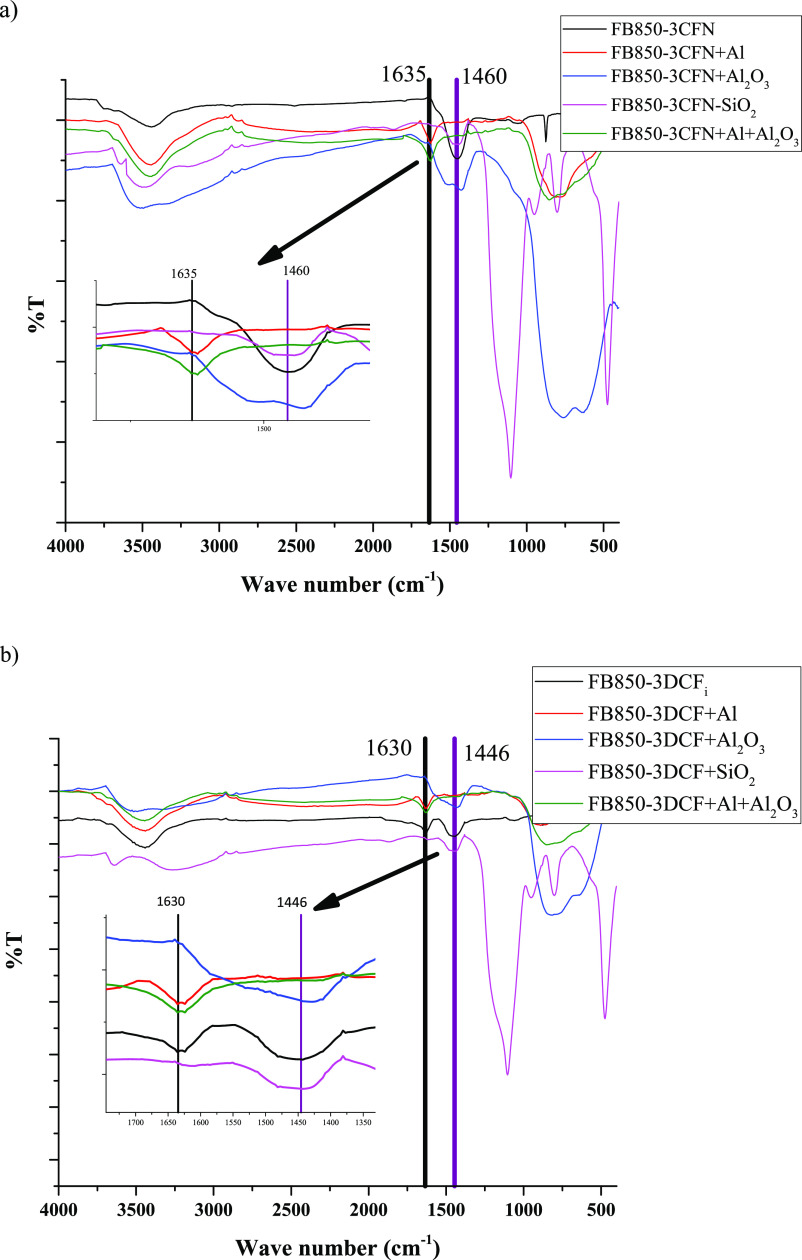
Infrared spectra of biochars grinding for 1 h: (a) caffeine and (b) diclofenac.

Figure 2 shows the infrared spectra of fique bagasse biochar solids at the end of each of the mechanochemical processes. These spectra showed changes in the signal at 1460 cm^–1^, which were assigned to the stretching of the C=N or C–N groups of caffeine or diclofenac, respectively. Therefore, it could be inferred that structural modifications occurred in both CFN and DCF with these treatments. However, the data were inconclusive. Consequently, HPLC-MS^*n*^ analyses were performed in order to identify the possible molecules formed as a consequence of the transformation suffered by CFN and DCF in each test.

**Figure 3:**
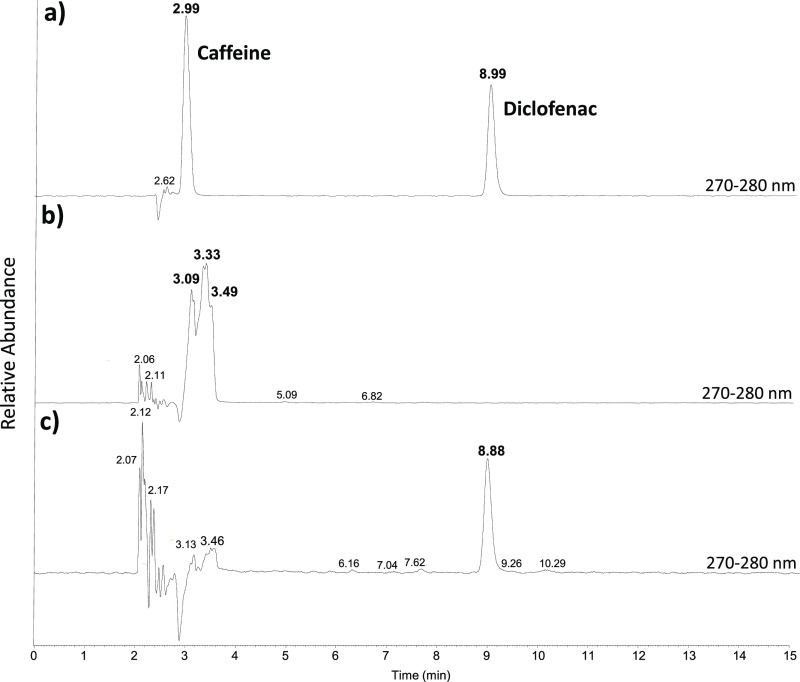
Chromatograms of the emerging contaminants studied. Chromatogram of a mixture of caffeine and diclofenac standards (a), Chromatogram of extracts obtained at the end of the mechanochemical process of the biochar with caffeine plus aluminum (FB850-3CFN-Al) (b) or diclofenac plus aluminum (FB850-3DCF-Al) (c). Other peaks with different retention times were observed in the chromatograms and evidenced the degradation compounds obtained from CFN and DCF.

The chromatograms obtained by HPLC-MS^*n*^ at the different times under evaluation did not show signals that evidenced the degradation of caffeine and diclofenac in the original biochar milling, as well as those that were mixed with the reagents: silicon oxide (SiO_2_) and aluminum oxide (Al_2_O_3_). However, the chromatograms of the extracts derived from the mechanochemistry samples obtained by using the biochar with caffeine or diclofenac plus aluminum (Al) (FB850-3CFN-Al and FB850-3DCF-Al) showed several UV absorption peaks at different retention times (Figure 3b,c) and different mass spectra. Meanwhile, the CFN and the DCF standards had one peak at the retention time of 2.99 and 8.99 min, respectively (Figure 3a). For this reason, this treatment was considered to be promising, and exhaustive studies of these results were subsequently carried out. In the case of caffeine, a significant degradation of the peak of this emerging contaminant was observed. A broader and nonsymmetrical peak was found at a longer retention time of 3.39 min (Figure 3b) compared to the observed in the reference standard (Figure 3a). In diclofenac, a decrease in the intensity of the compound peak was observed upon analysis of the chromatogram baseline by comparison with the solvent front (Figure 3c).

**Figure 4:**
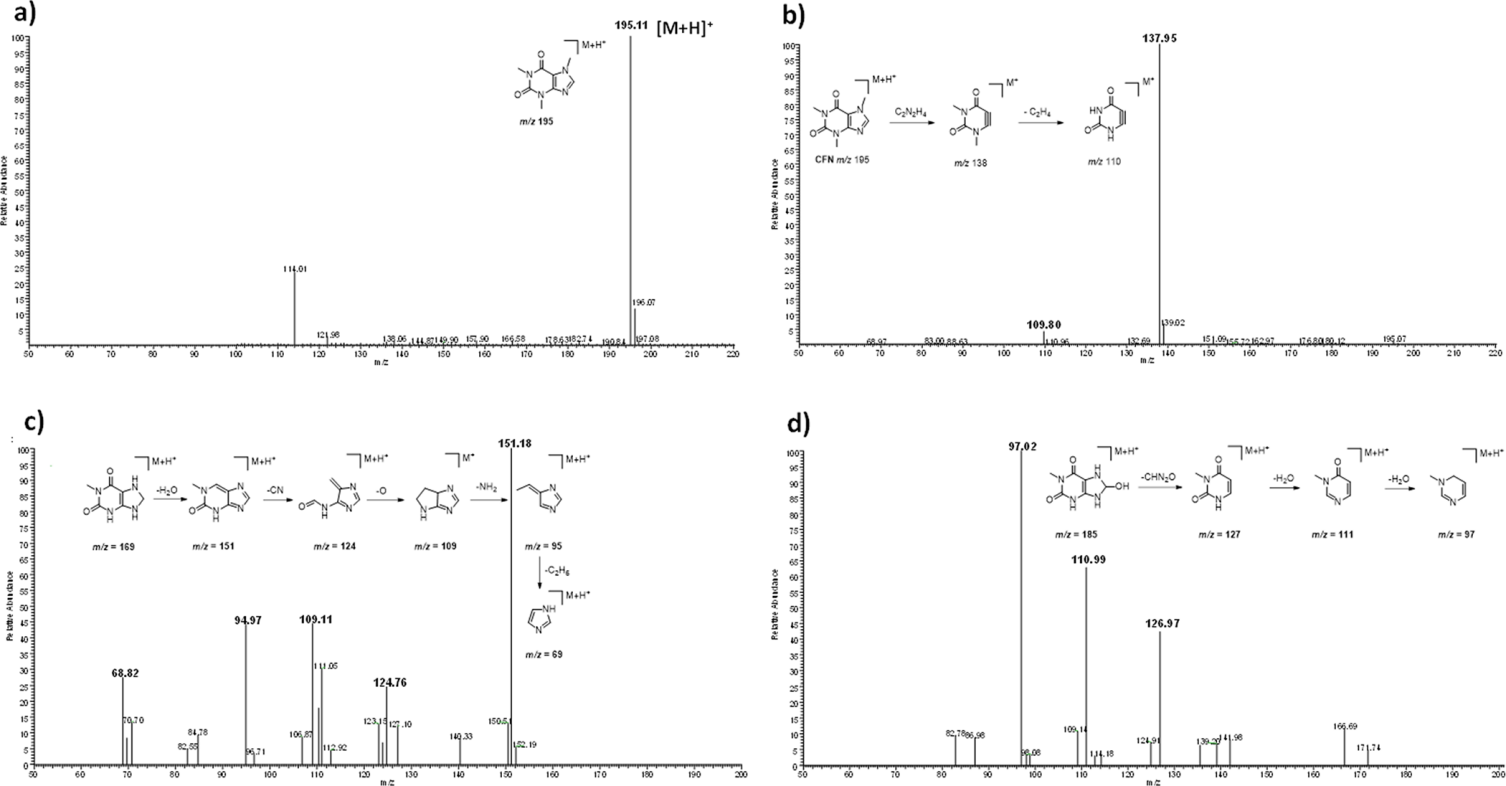
Mass spectra of CFN and its degradation products. (a) Full MS spectrum of CFN, (b) MS/MS spectrum of CFN, (c) MS/MS spectrum of compound C3, and (d) MS/MS spectrum of compound C6.

Figure 4 and Table 1 show the chemical structures proposed for products derived from the mechanochemical-Al transformation of CFN. In order to elucidate these molecules, CFN fragmentation was studied first. The protonated ion [M + H]^+^ at *m*/*z* 195 detected (Figure 4a) was the precursor of fragments *m*/*z* 138 and 110 (Figure 4b). These ions were the result of the loss of C_2_H_4_NO and C_2_H_6_, respectively.^21,22^Figure 4c,d displays the mass spectra of possible caffeine derivatives elucidated after our experiments. For instance, in each of the mass spectra studied, the molecular ion [M + H]^+^ was identified, and then, the structures of the transformation products were established by following the fragmentation pattern (Figures 4c,d and S1). In total, eight CFN mechanochemical-Al transformation compounds were identified. All of these compounds had a longer retention time than CFN; therefore, it can be deduced that they are less polar than CFN. The higher affinity to the stationary phase (C18 column) indicates an apolar behavior of part of the analytes.

**Table 1:** Compounds Identified by HPLC-ESI-MS^*n*^ upon the Degradation of CFN

compound	retention time (min)	[M + H]^+^ (*m*/*z*)	MS/MS product ions (*m*/*z*)	references
CFN	3.04	195.09	138, 110	(21,22)
C_1_	3.33	183.17	165, 134, 122, 100, 83	(23)
C_2_	3.99	182.97	165, 137, 127, 109	(23)
C_3_	4.16	169.03	151, 124, 109, 95, 69	(23)
C_4_	5.09	142.89	125, 124, 120, 117, 115	(24−26)
C_5_	5.74	143.05	125, 124, 120, 115	(27,28)
C_6_	6.82	185.07	167, 142, 127, 111, 97, 87	(23,24,26,27)
C_7_	10.13	225.08	207, 183, 167, 127, 113, 97	(24,25)
C_8_	11.28	165.05	125, 120, 117, 115	(23)

**Figure 5:**
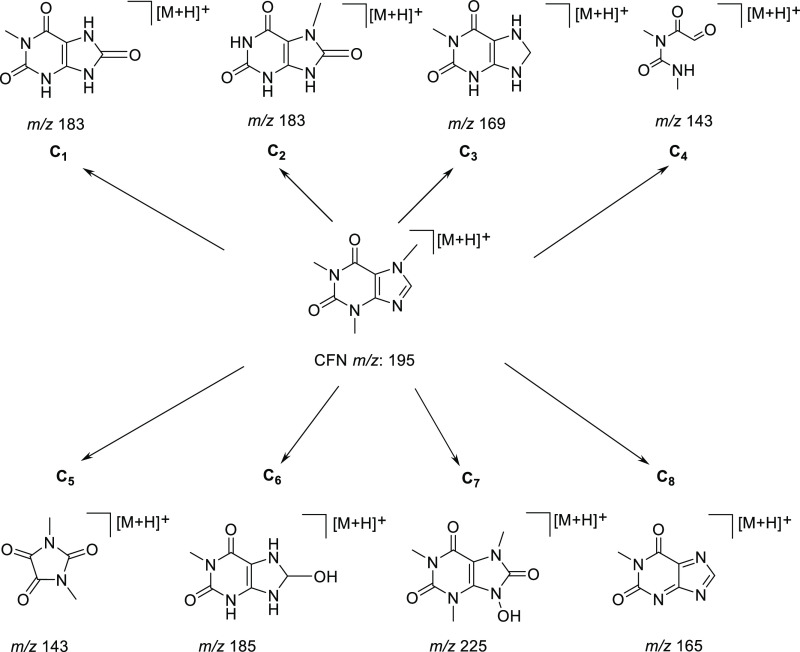
Proposed molecules after the mechanochemical transformation of caffeine.

Our results suggested the presence of eight CFN mechanochemical-Al transformation products, which were named C1–C8 (Figures 5 and S1). The proposed caffeine mechanochemical-Al transformation for C1, C2, C6, and C7 showed that hydroxyl radical (^·^OH) could attack C8=N9 bonds of the 5-member ring to prompt the addition of hydroxyl (−OH) and subsequently hydroxyl oxidation to the carbonyl group for C1, C2, and C7.^23−26,29^ Afterward, the presence of the carbonyl group on C8=N9 could cause the opening the five-member ring, and the following hydroxyl radical (^·^OH) could attack the C5=C6, leading to the formation of C4 and C5.^24−26,28,29^ In addition, molecules C1, C2, C3, C6, and C8 generated *N*-demethylation (Figures 5 and S1). This reaction has been suggested as the pathway for the degradation of caffeine by *Paraburkholderia caffeinilytica* and many microorganisms.^30^

According to the literature, in the past few years, several strategies for reducing caffeine degradation have been studied. For instance, microbial catalysis,^27^ gen bacterial cluster,^30^ UV/chlorination,^31−37^ photo-Fenton,^38^ ozonation,^24,39^ UV photolysis,^40^ photocataytic degradation,^41^ and semiconductor-based heterogeneous photocatalysis^42,43^ were found to be effective in eliminating caffeine. It is interesting to note that in many studies, hydroxyl radicals were found to be the most important radical for the degradation of CFN.^29^ For example, Jia showed that ^·^OH and SO_4_^·–^ were the main radicals generated using peroxydisulfate and peroxymonosulfate in the presence of Mn_2_O_3_, and these radicals could contribute to the degradation of CFN.^44^

In another related study, the use of composites of NiO/TiO_2_-F and CuO/TiO_2_-F under UV irradiation was reported to result in caffeine degradation. This process is the result of hydroxyl radicals forming a C8–OH radical adduct that forms 8-oxocaffeine and subsequently 1,3,7-trimethyluric acid as major side-products. This result was supported by theoretical studies.^45^ Additionally, Prado, performed and monitored the photoelectrocatalytic degradation of caffeine using bismuth vanadate modified with reduced graphene oxide (BiVO_4_/rGO). The most striking result to emerge from these data is that the hydroxyl radical resulted in caffeine degradation.^42^ Moreover, the photocatalytic activity of a composite of Fe_2.5_Co_0.3_Zn_0.2_O_4_ and copper–chromium layered double hydroxide (CuCr-LDH) was evaluated. The results obtained showed higher production and better transference of electrons; consequently, the production of more hydroxyl radicals was demonstrated.^46^

**Figure 6:**
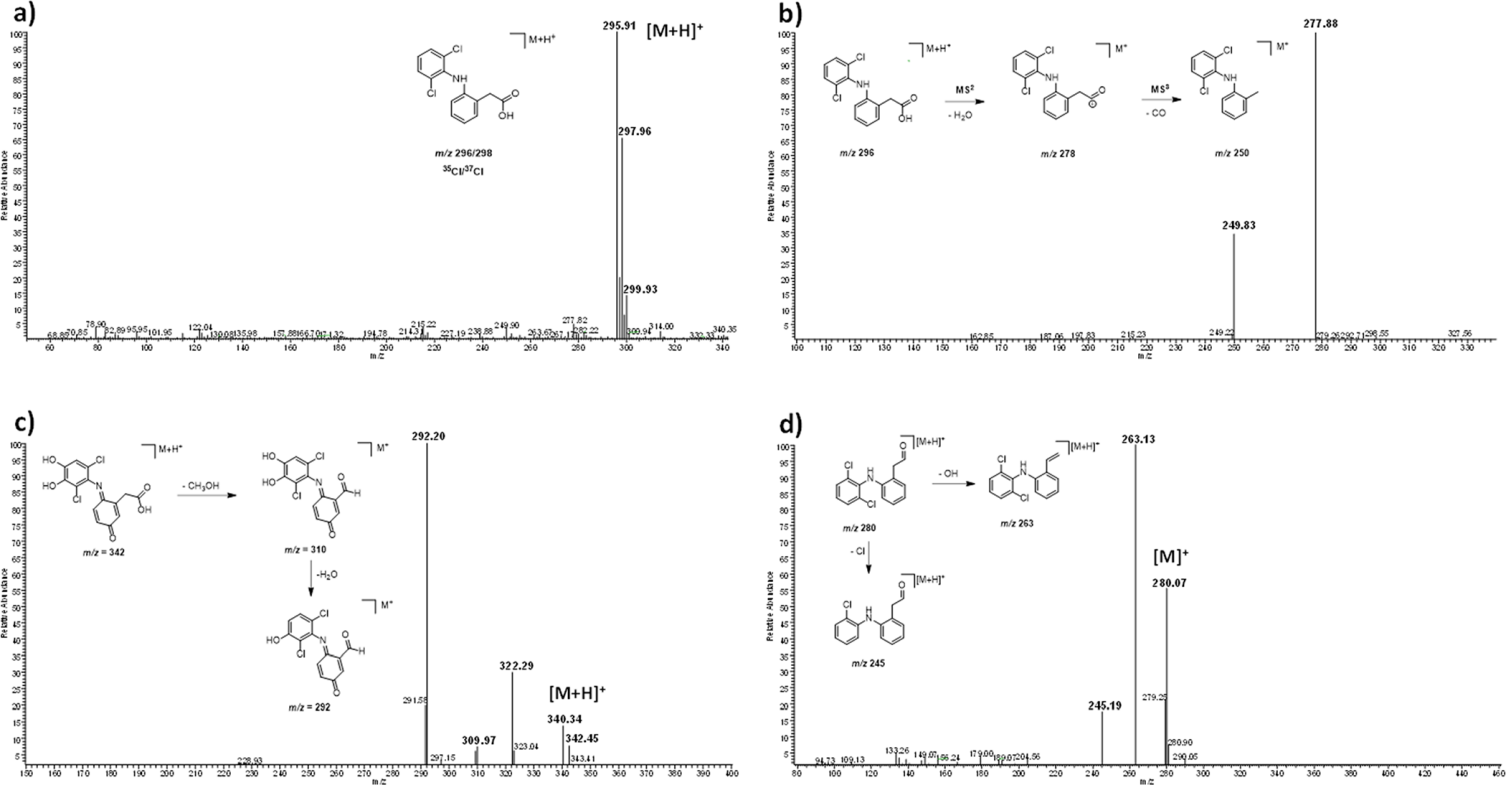
Mass spectra DCF and its degradation products. (a) Full MS spectrum of DCF, (b) MS^3^ spectrum of DCF, (c) MS/MS spectrum of compound D3, and (d) MS/MS spectrum of compound D7.

**Figure 7:**
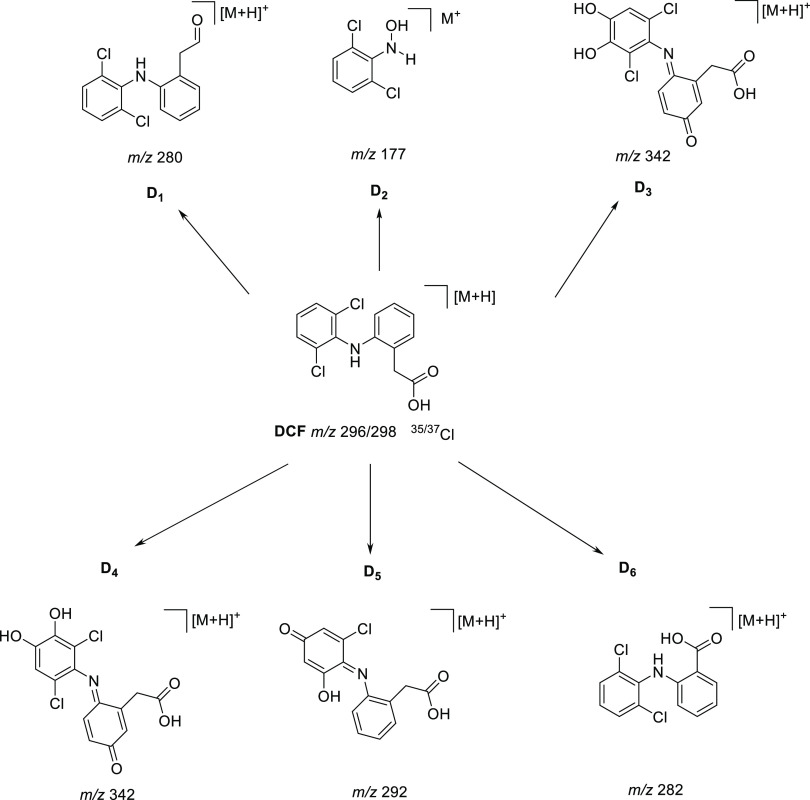
Suggested molecules after the mechanochemical transformation of diclofenac.

Furthermore, Figure 3 shows that the DCF solution had one peak at a retention time of 8.99 min, while samples after mechanochemical-Al transformation give an absorption peak at the same retention time and additional peaks. It demonstrated DCF transformations produced by this procedure. Figures 6, 7 and Table 2 display the chemical structures suggested for products derived from mechanochemical-Al transformation of DCF and Figure S2 shows mass spectra of possible molecules elucidated after our experiments. Previously, to elucidate these molecules, DCF fragmentation was studied. In fact, mass spectra of diclofenac displayed the predominant^35^ Cl_2_/^12^C isotope at *m*/*z* 296 with the molecular ions (M+, M + 2y M + 4), and the isotopic cluster was clearly shown with relative intensities in ratios of 9:6:1 (Figure 6a). Tandem MS permitted elucidate the characteristic DCF fragmentation, and the MS/MS spectrum shows a peak at *m*/*z* 278 [M + H-18] which indicates the loss of H_2_O, while MS^3^ presents one peak to *m*/*z* 250 [M + H-18–44] corresponding to the loss of CO_2_ consecutive to the loss of initial water (Figure 6b) and finally loses HCl to give fragments at *m*/*z* 215 was evidenced in MS^4^. Successive fragmentation is obtained in the characterization of DCF permitted to confirm the typical ions for this compound.^47−50^

**Table 2:** Summary of Identified Intermediates Determined by HPLC/ESI-MS upon Transformation of DCF

compound	retention time (min)	[M + H]^+^ (*m*/*z*)	MS/MS product ions (*m*/*z*)	references
DCF	8.99	295.91/297.96	278, 250, 215. **MS**^**3**^(278): 250, 215. **MS**^**4**^(250): 215, 214	(47−50)
D_1_	6.16	280.07	263, 245, 179, 133	(51−55)
D_2_	7.62	176.80	158, 149	(49,51,52,56,57)
D_3_	8.48	342.45	322, 310, 292	(52,53,55−58)
D_4_	9.26	341.95	322, 310, 292	(52,53,55−58)
D_5_	10.29	292.13	275, 194, 156	(56)
D_6_	13.59	282.09	267, 245, 163, 142	(51−55)

It is interesting to note that the HPLC-MS^*n*^ analysis displayed six peaks that correspond to possible products of mechanochemical-Al transformation of DCF. These products were assigned D1–D6 (Figure 7). Elucidation of the structures and MS fragments of all products is shown in Figure 7 and Table 2. Overall, the intermediate product with *m*/*z* 312 molecular ion was assigned as a monohydroxylated compound, while *m*/*z* 332 was a multihydroxylated compound. Intermediate *m*/*z* 342 belonged to multihydroxylated and quinoid-type compound, whereas the *m*/*z* 292 was monohydroxylated and quinoid-type compound (Figure 6c); in addition, the product with *m*/*z* 280 molecular ion was assumed to be a product monohydroxylated and decarboxylated (Figure 6d), whereas the *m*/*z* 177 revealed cleavage of C–N bonds to produce other intermediates may result in the generation of aromatic products with one benzene (Figure S2).^48,54^

Many researchers have described the degradation of diclofenac sodium using various processes, such as photocatalysis by TiO_2_,^59^ UV-/H_2_O_2_,^60^ heterogeneous-FeCeOx Fenton,^61^ ultrasound intensified with FeCeOx,^62^ gamma-irradiation induced degradation,^63^ and sonoelectrochemical degradation.^64^ These results are significant for the fact that evidence that reactive species (hydroxyl radical, ^·^OH, sulfate radical, SO_4_^·–^, superoxide radical anion, ^·^O_2_^–^, and singlet oxygen, ^1^O_2_) are important molecules for diclofenac remotion.^57^ As an example, in the degradation of diclofenac employing persulfate anions activated through ultrasound, it was exposed that hydroxyl and sulfate radicals were the major species involved in the DCF degradation.^52^ On the other hand, using a hybrid material of carbon quantum dots and BiOCOOH for photocatalytic degradation of DCF, it was evidenced that the most important reactions in the process were e^–^ reduction, ^·^O_2_^–^ attack, and ^·^OH additions.^65^ Additionally, research using a nanocomposite of graphene oxide, Ag, and BiOI for photodegradation of diclofenac showed that the decomposition of DCF was possible by the generation of ^·^OH and ^·^O_2_^–^ radicals.^66^ Furthermore, studies with the H_2_O_2_-assisted photoelectrocatalytic degradation of diclofenac with the g-C_3_N_4_/BiVO_4_ composite demonstrated that the main active species for the DCF degradation was ^·^OH and ^·^O_2_^–^.^49^

Additionally, the finding determined by this research validates the usefulness of mechanochemical processes to enable the formation of species with enhanced reactivity and stability to drive solid-phase reactions.^67^ What is known, reactive oxygen species (ROS) like ^·^OH are necessary for advanced oxidation processes, and ROS could be generated through the activation of radical precursors by the catalytic processes.^42,44^ Actually, molecular oxygen is a green oxidant, however, because of the spin-forbidden nature of the O_2_ molecule; it can barely degrade pollutants by oxidation under mild circumstances.^68^ Therefore, it is necessary to work with methods to obtain ROS using molecular oxygen. For instance, zerovalent metals (ZVMs) like zerovalent zinc (ZVZ), zerovalent copper (ZVC), zerovalent iron (ZVI), and zerovalent aluminum (ZVAl) have been widely employed as heterogeneous catalysts to activate molecular oxygen to generate ROS.^68−70^ Among these, ZVAl possesses lower standard redox potential (E0(Al^3+^/Al) = −1.667 V), which offers a greater thermodynamic driving force for electron transfer compared with the other ZVMs.^69,71−73^ Nevertheless, ZVAl is always covered with a layer of oxide film under ambient conditions, which inhibits the electron release from its surface.^74^

Therefore, it is required to destroy the surface oxide films, and this is possible using high-energy ball milling (also called mechanical activation).^75^ The ZVAl/air system employed in a mechanochemical condition could produce the ^·^OH formation. The processes that may have occurred during the mechanochemical aerobic treatment of aluminum powder are described as the following eqs 1–5. First, corrosion of the surface oxide films of ZVAl, through mechanical activation, releases electrons and produces Al^3+^; subsequently, molecular oxygen reduction could be produced ^·^O_2_^–^ (eqs 1 and 2). Afterward, ^·^O_2_^–^ forming the radicals HO_2_^·^ and H_2_O_2_ are in-situ-generated due to the rapid disproportionation of HO_2_^·^ (eqs 3 and 4). Afterward, H_2_O_2_ could be reduced by ZVAl to produce ^·^OH (eq 5).^68,71,76,77^
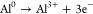
1
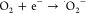
2
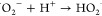
3
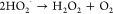
4
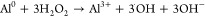
5

As proposed by the previous findings in the literature, the most important radical for the degradation of organic contaminants is ^·^OH, which is nonselective and could be oxidized and mineralized by almost all kinds of organic molecules.^78^ In addition, our results have a number of similarities with previous results reported in the literature in which it is argued that intermediates are usually generated during the degradation of organic contaminants.^53^

## 4 Conclusions

In this study, a fique bagasse biochar was used for the removal of caffeine and sodium diclofenac from an aqueous solution; after that, the degradation of CFN and DCF through the mechanochemical/coreactants process was investigated. The results suggested that mechanochemical degradation of CFN and DCF was needed to start the reaction; in addition, significant influences of the coreactant type employed for the degradation process were evidenced. Actually, it was found that the CFN and DCF mechanochemical degradation was more effective in the presence of Al. Furthermore, mass spectrometry allowed us to demonstrate the different transformation products of CFN and DCF proposed in this work due to hydroxylation, decarboxylation, dehydration, demethylation, C–C bonding, and C–N bonding hydroxylation, decarboxylation, dehydration, demethylation, C–C bonding, and C–N bonding. Although the result showed limitations in several ways, for example, experiments to determine the existence of radical species and theoretical calculations that allow inferring degradation pathways of CFN and DCF through the mechanochemical process are necessary, this approach has the potential for further research in order to evaluate diverse parameters to optimize the mechanochemical treatment process for pollutants.

## Supporting Information Available

MS spectra for intermediate products identified of mechanochemical treatment of fique bagasse biochar with caffeine and aluminum; MS spectra for intermediate products identified of mechanochemical treatment of fique bagasse biochar with diclofenac and aluminum (PDF)
