# Supplementary material for: Mechanochemical Degradation of Caffeine and Diclofenac Using Biochar of Fique Bagasse in the Presence of Al: Monitoring by Mass Spectrometry
Source: ACS Omega. 2023 Oct 12;8(42):38905–15. doi: 10.1021/acsomega.3c03051 (PMC10601424; doi:10.1021/acsomega.3c03051)
Supplement: Supplementary file 1 — ao3c03051_si_001.pdf [file ao3c03051_si_001.pdf]

Supporting information

Mechanochemical degradation of caffeine and diclofenac using biochar of fique bagasse in the presence of Al: Monitoring by mass spectrometry.

Yaned Milena Correa-Navarro[a,b], Gerson-Dirceu López[c], Chiara Carazzone[d], Liliana Giraldo[e], and Juan Carlos Moreno-Piraján[b*]

[a]Departamento de Química, Facultad de Ciencias Exactas y Naturales. Grupo de investigación Estudios Ambientales en Agua y Suelo, Universidad de Caldas, Manizales 170004, Caldas, Colombia.
[b]Departamento de Química, Facultad de Ciencias, Grupo de investigación en Sólidos Porosos y Calorimetría, Universidad de los Andes, Carrera 1 No. 18 A-12, Bogotá D.C. 111711, Colombia.

[c]PhysCheMath Research Group, Facultad de Ciencias y Humanidades, Universidad de América, Avda. Circunvalar No. 20-53, Bogotá D.C. 111711, Colombia.

[d]Laboratory of Advanced Analytical Techniques in Natural Products (LATNAP), Departamento de Química, Facultad de Ciencias, Universidad de los Andes, Carrera 1 No. 18 A-12, Bogotá D.C. 111711, Colombia.

[e]Departamento de Química, Facultad de Ciencias, Universidad Nacional de Colombia, Sede Bogotá, Bogotá D.C. 11001, Colombia.

*E-mail corresponding author: jumoreno@uniandes.edu.co

Keywords

Ball mill, emerging contaminants, remediation, Aluminum-based composite, molecular oxygen activation.

Figure S1. MS spectra for intermediates products identified of mechanochemical treatment of fique bagasse biochar with caffeine and Aluminum.

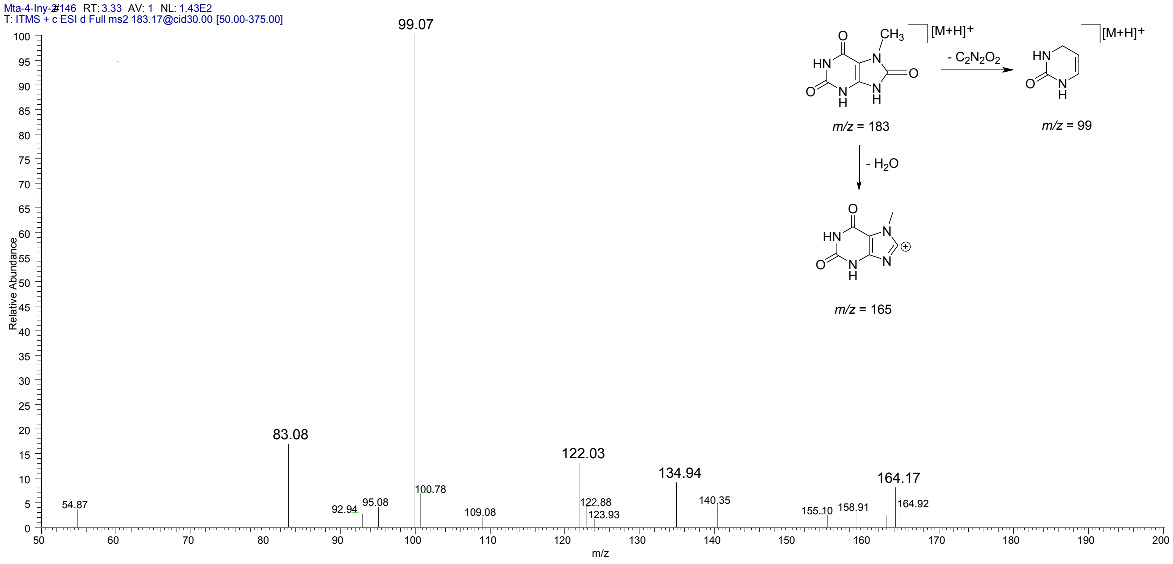

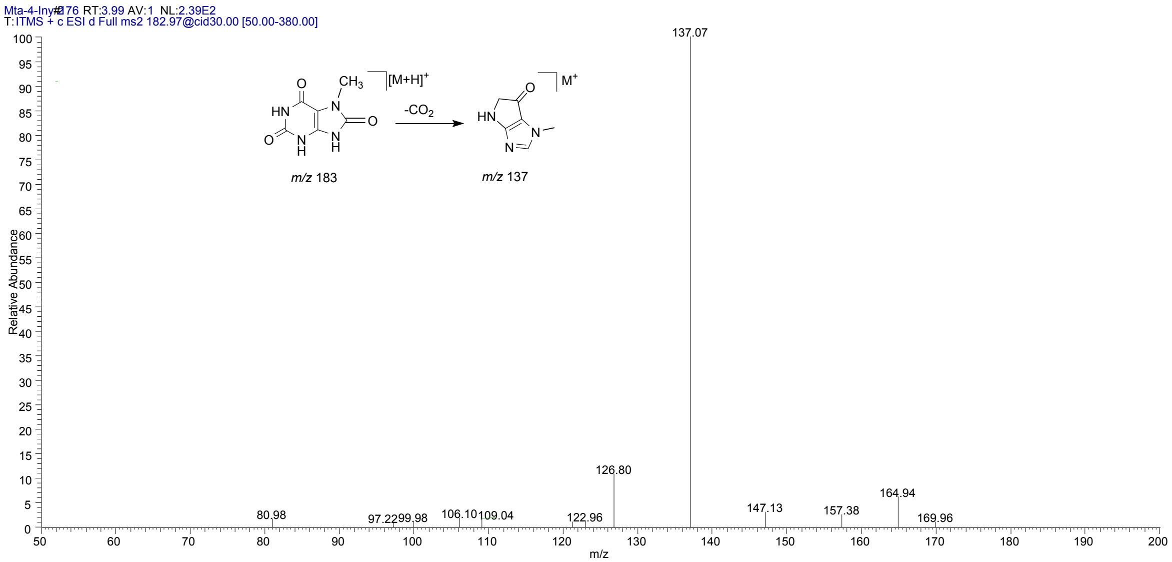

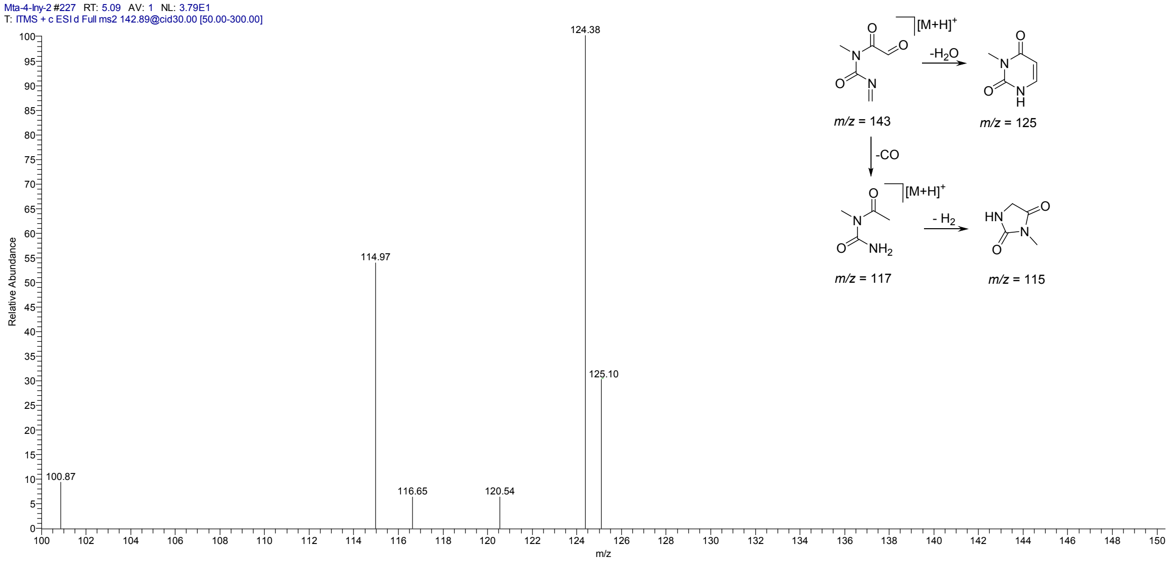
Mta-4-Iny-2 #227 RT: 5.09 AV: 1 NL: 3.79E1
T: ITMS + c ESI d Full ms2 142.89@cid30.00 [50.00-300.00]
Relative Abundance
m/z
124.38
114.97
125.10
100.87
116.65
120.54
[M+H]+
-H2O
m/z = 143
m/z = 125
-CO
- H2
m/z = 117
m/z = 115

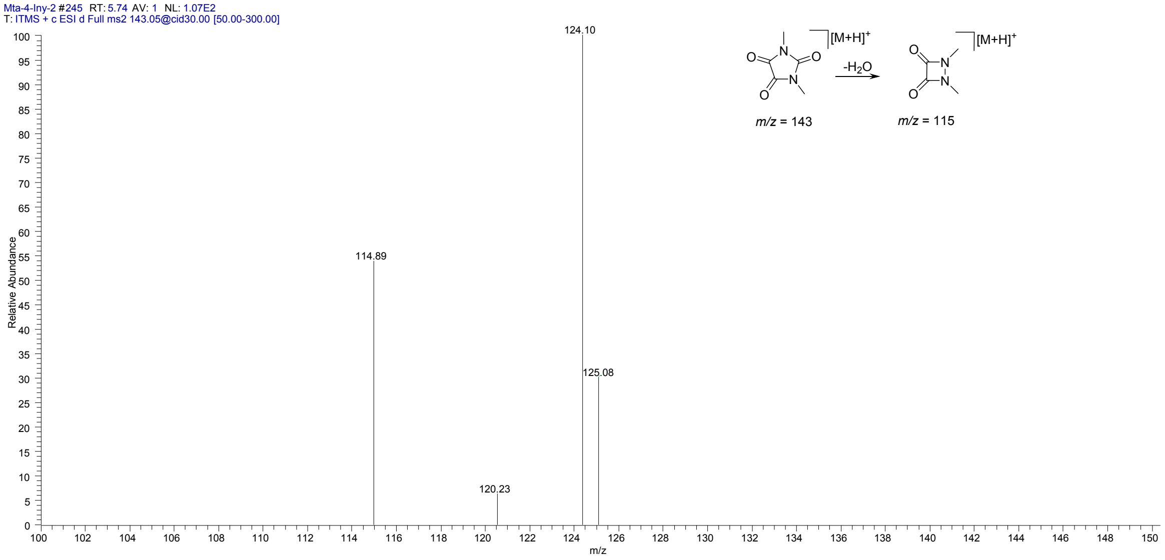
Mta-4-Iny-2 #245 RT: 5.74 AV: 1 NL: 1.07E2
T: ITMS + c ESI d Full ms2 143.05@cid30.00 [50.00-300.00]
Relative Abundance
m/z
124.10
114.89
125.08
120.23
[M+H]+
-H2O
m/z = 143
m/z = 115

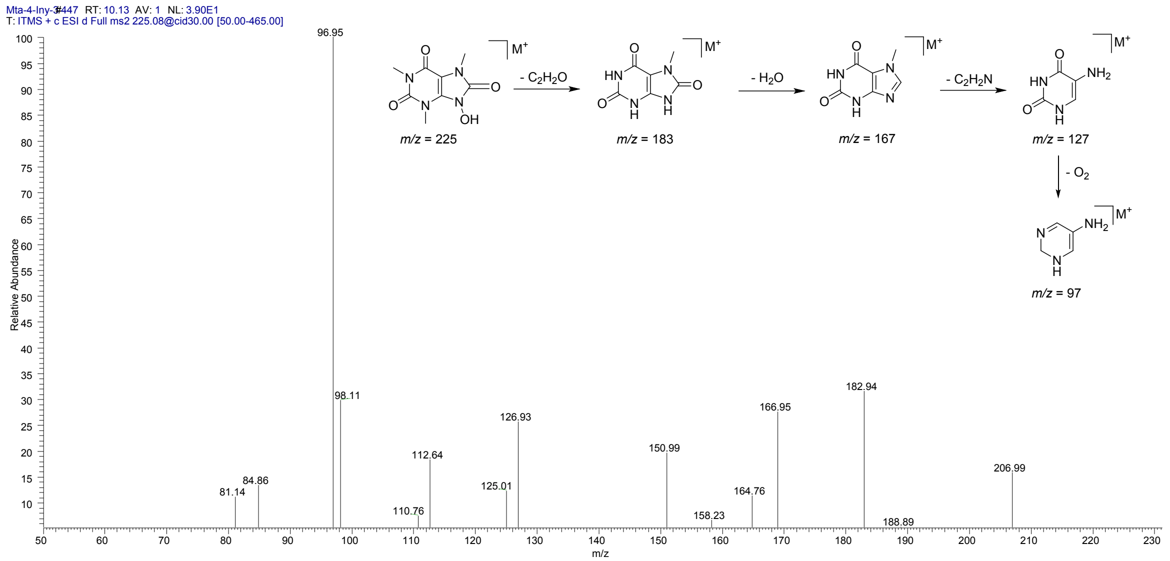
Mta-4-lny-3#447 RT: 10.13 AV: 1 NL: 3.90E1
T: ITMS + c ESI d Full ms2 225.08@cid30.00 [50.00-465.00]
Relative Abundance
m/z
96.95
98.11
81.14
84.86
110.76
112.64
125.01
126.93
150.99
158.23
164.76
166.95
182.94
188.89
206.99
- C2H2O
- H2O
- C2H2N
- O2
m/z = 225
m/z = 183
m/z = 167
m/z = 127
m/z = 97

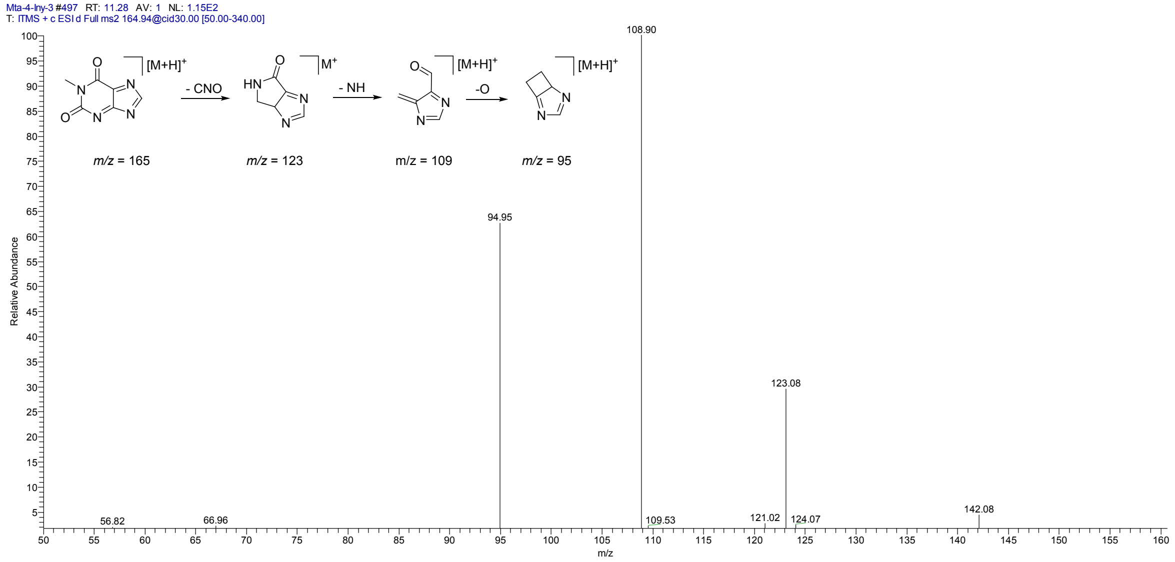
Mta-4-lny-3 #497 RT: 11.28 AV: 1 NL: 1.15E2
T: ITMS + c ESI d Full ms2 164.94@cid30.00 [50.00-340.00]
Relative Abundance
m/z
108.90
94.95
123.08
56.82
66.96
109.53
121.02
124.07
142.08
- CNO
- NH
-O
m/z = 165
m/z = 123
m/z = 109
m/z = 95

Figure S2. MS spectra for intermediates products identified of mechanochemical treatment of fique bagasse biochar with diclofenac and Aluminum.

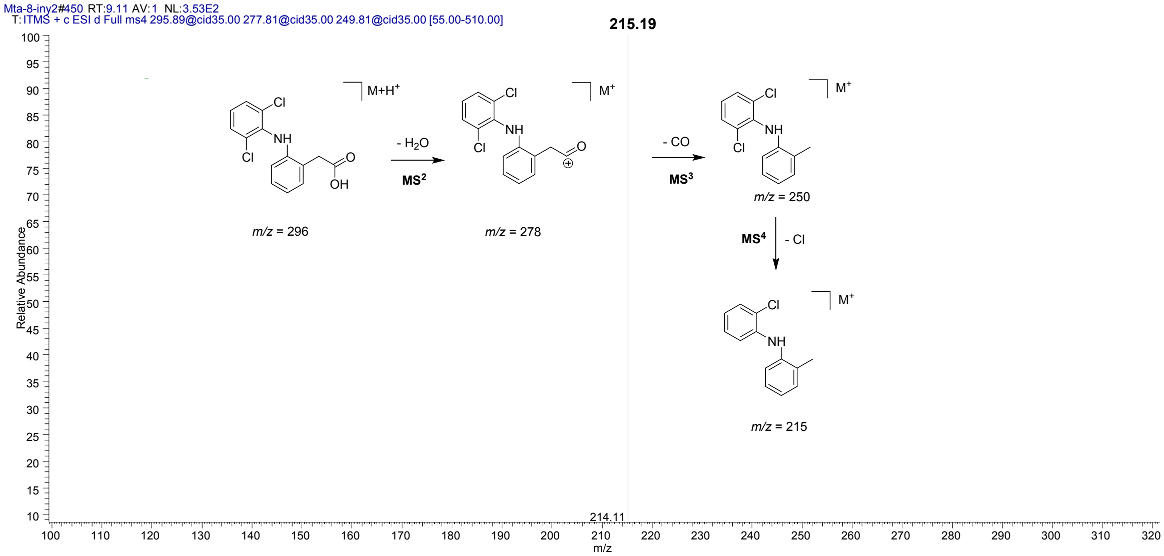

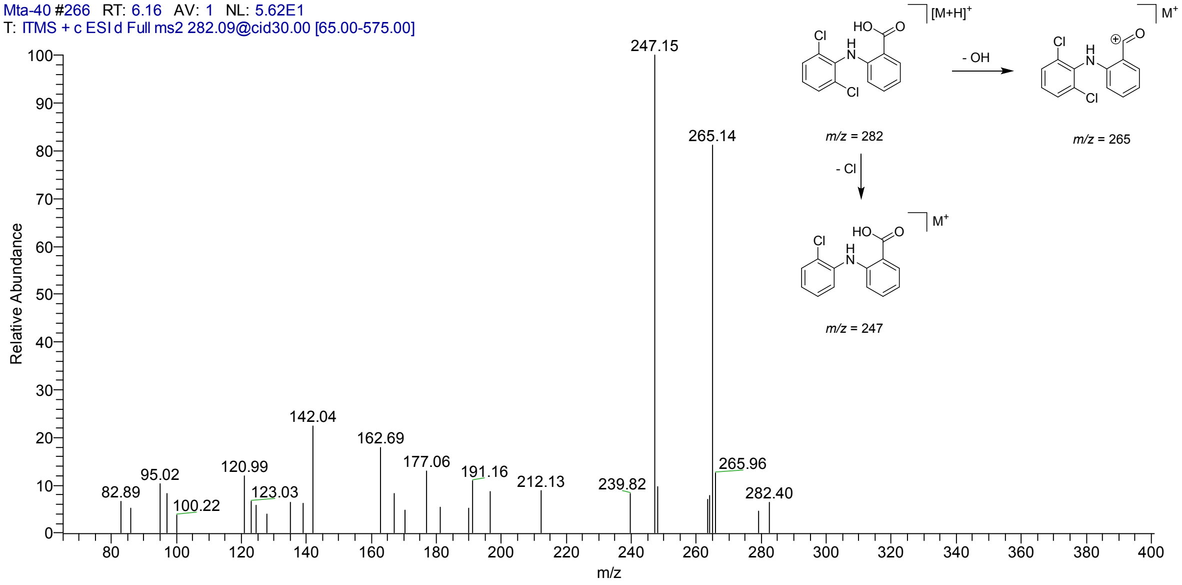

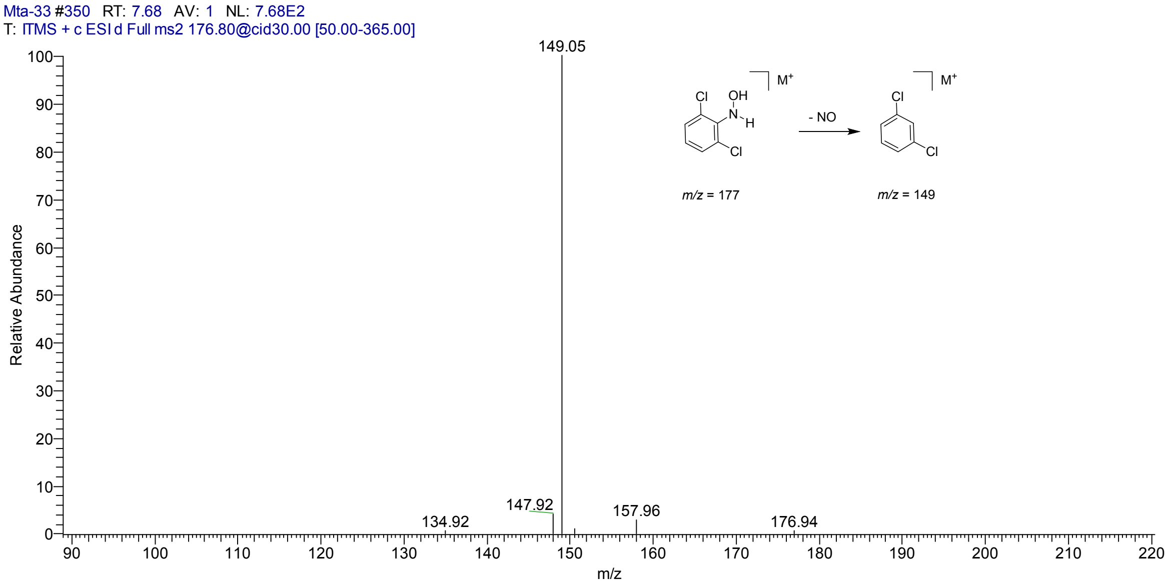

Mta-33 #350 RT: 7.68 AV: 1 NL: 7.68E2
T: ITMS + c ESI d Full ms2 176.80@cid30.00 [50.00-365.00]
149.05
147.92
134.92
157.96
176.94
Relative Abundance
m/z
M+
- NO
m/z = 177
m/z = 149

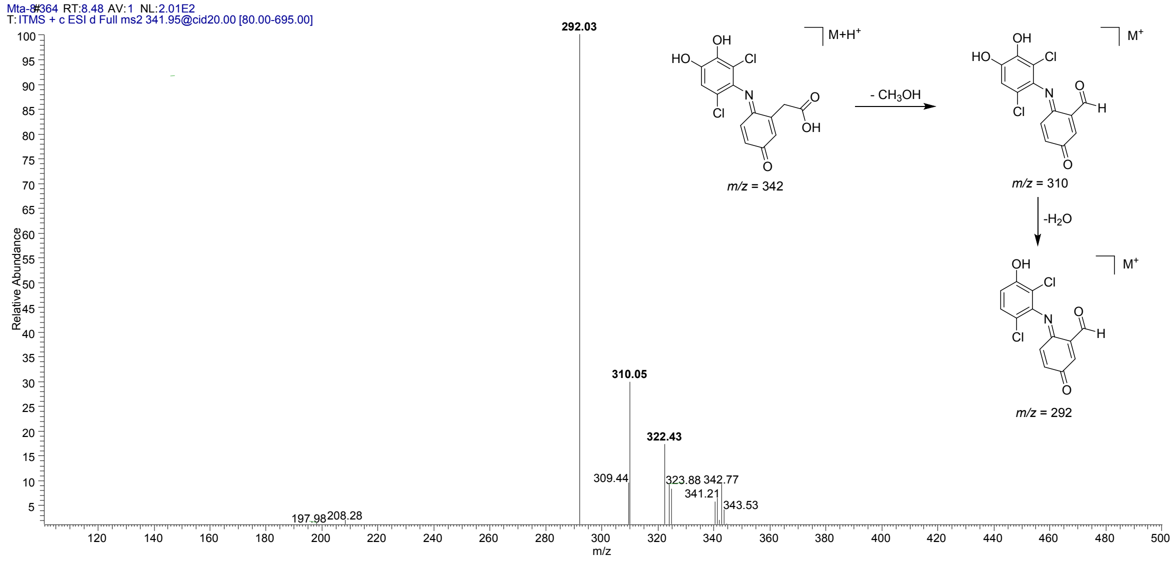

Mta-8#364 RT:8.48 AV:1 NL:2.01E2
T: ITMS + c ESI d Full ms2 341.95@cid20.00 [80.00-695.00]
292.03
310.05
322.43
309.44
323.88
342.77
341.21
343.53
197.98
208.28
Relative Abundance
m/z
M+H+
- CH3OH
m/z = 342
m/z = 310
-H2O
m/z = 292

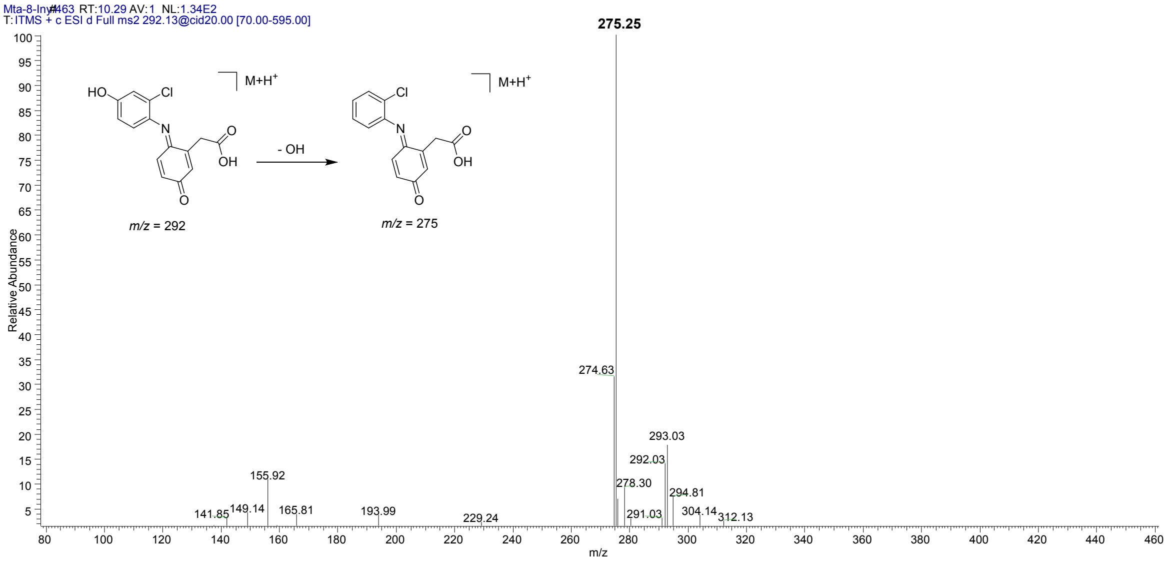

Mta-8-Inv#463 RT:10.29 AV:1 NL:1.34E2
T: ITMS + c ESI d Full ms2 292.13@cid20.00 [70.00-595.00]
275.25
M+H+
HO
Cl
N
O
OH
O
- OH
m/z = 292
m/z = 275
Relative Abundance
274.63
293.03
292.03
278.30
294.81
155.92
141.85
149.14
165.81
193.99
229.24
291.03
304.14
312.13
m/z
